# Characterization of a novel comprehensive genomic profiling test with better detection of heterozygous deletions and RNA-based gene fusion analysis

**DOI:** 10.1093/oncolo/oyaf056

**Published:** 2025-08-11

**Authors:** Ryouta Kakuta, Akira Takemoto, Kousuke Tanimoto, Rika Noji, Ryo Kudo, Yoshihito Kano, Yayoi Ando, Tatsunori Simoi, Yusuke Kinugasa, Sadakatsu Ikeda

**Affiliations:** Department of Precision Cancer Medicine, Tokyo Medical and Dental University Hospital, Tokyo, Japan; Department of Gastrointestinal Surgery, Tokyo Medical and Dental University, Tokyo, Japan; Disease Biorepository Center, Tokyo Medical and Dental University, Tokyo, Japan; Research Core, Institute of Research, Tokyo Medical and Dental University, Tokyo, Japan; Department of Precision Cancer Medicine, Tokyo Medical and Dental University Hospital, Tokyo, Japan; Department of Oral Surgery, Tokyo Medical and Dental University Hospital, Tokyo, Japan; Department of Precision Cancer Medicine, Tokyo Medical and Dental University Hospital, Tokyo, Japan; Department of Surgery, Kyoto Katsura Hospital, Kyoto, Japan; Department of Precision Cancer Medicine, Tokyo Medical and Dental University Hospital, Tokyo, Japan; Department of Clinical Oncology, Tokyo Medical and Dental University Hospital, Tokyo, Japan; Clinical Research Support Office, Research Management Division, National Cancer Center Hospital, Tokyo, Japan; Department of Medical Oncology, National Cancer Center Hospital, Tokyo, Japan; Department of Gastrointestinal Surgery, Tokyo Medical and Dental University, Tokyo, Japan; Department of Precision Cancer Medicine, Tokyo Medical and Dental University Hospital, Tokyo, Japan; Moores Cancer Center, University of California, San Diego, USA

**Keywords:** cancer genomic profiling, RNA-based gene fusion analysis

## Abstract

Cancer genomic profiling has revolutionized cancer research and clinical practice by facilitating personalized medicine approaches. Next-generation sequencing technologies have played a pivotal role in expanding the possibilities of cancer genomics, providing a comprehensive view of the genomic landscape of tumors and identifying potential therapeutic targets. In Japan, the approval of several cancer-related gene panel tests has accelerated the implementation of cancer genomic medicine. However, there remains a need for evidence-based selection criteria for the different panel tests available. Among these tests, ACTOnco+® stands out as a comprehensive cancer genomic profiling tool. In this study, we compared 110 samples from 29 different tumor types using FoundationOne® CDx (324 genes for DNA analysis) and ACTOnco+® (440 genes for DNA and 31 genes for RNA analysis). Overall, the mutation profiles between the 2 assays exhibited 82.8% positive agreement in reported sequence alterations in clinically actionable genes, including single nucleotide variants and insertions–deletions. Copy number gains showed concordance of 76.9%, and copy number losses in 66.7%. In the case of KIAA1549-BRAF fusion-positive astrocytoma, the fusion event was not detected by DNA only test, but RNA sequencing identified the rearrangement. The patient exhibited clinical benefit from MEK inhibitor treatment. Tumor mutational burden and microsatellite instability (MSI) demonstrated high concordance across various cancer types. ACTOnco+® identified a total of 329 heterozygous deletions, which were subsequently validated for reliability using FISH analysis. Gene profiling with ACTOnco+® exhibited comparable results to FoundationOne® CDx, thereby contributing to future personalized medicine endeavors.

**Implications for Practice**

Our research provides a head-to-head comparison of 2 cancer genomic profiling platforms. Through the analysis of 110 samples spanning 29 different tumor types, our study not only assesses the concordance between these platforms but also highlights the clinical implications of genomic alterations detected. Significantly, the study underscores the importance of RNA analysis in identifying therapeutically actionable targets, as evidenced by a case of KIAA1549-BRAF fusion-positive astrocytoma, which was only detected through RNA sequencing.

## Background

In recent years, advancements in next-generation sequencing (NGS) technologies have facilitated the implementation of clinical sequencing as part of precision cancer medicine.^1,2^ In the United States, the Precision Medicine Initiative, initiated in 2015, focuses on detecting driver gene abnormalities in individual patients’ cancer tissues and using evidence-based approaches to select and administer targeted anticancer therapies.^3^ The National Comprehensive Cancer Network has incorporated genetic analysis of cancer into its guidelines, recommending it as part of standard care for cancers.^4-6^ Cancer gene panel testing, which focuses on analyzing approximately 100-400 cancer-related genes, has been widely used with several testing companies actively developing such panels.

In Japan, since June 2019, the NCC Oncopanel® and FoundationOne® CDx have been approved, promoting the implementation of precision cancer medicine.

Patients who receive treatment tailored to their biomarkers have shown improved survival rates compared to those who receive non-matched treatments.^7^ Furthermore, comprehensive NGS assays allow for the simultaneous detection of multiple genomic alterations (SNVs, indels [insertions–deletions], rearrangements, copy number [CN] alterations, microsatellite instability [MSI], Tumor mutation burden [TMB]) from a single patient sample, enabling the acquisition of more information from limited samples. TMB, reported as the number of DNA mutations per megabase (Muts/MB), serves as an indicator of the overall mutation burden in a tumor. High TMB has been associated with favorable responses to immune checkpoint inhibitors. As a result of accumulating clinical utility, cancer genomic profiling is predicted to become the standard of care in cancer treatment.

ACTOnco+® is one of the cancer genomic profiling assays, offering a comprehensive cancer panel capable of systematically interrogating 440 cancer-related gene mutations. In addition to analyzing DNA, ACTOnco+® distinguishes itself by incorporating RNA-based gene fusion assay and the reporting of heterozygous deletions for haploinsufficient genes that are potentially actionable.

Despite the existence of various types of cancer genomic profiling assays, there is limited data available in the clinical supporting the selection of specific panel tests. In this study, we aimed to compare the gene mutation detection rates and the number of clinically relevant gene mutations identified between ACTOnco+® and FoundationOne® CDx. By elucidating the differences in the detection of mutated genes and the identification of treatment options, our objective is to advance more precise personalized medicine strategies.

## Methods

### Ethical considerations

The study was conducted in accordance with the principles outlined in the Helsinki Declaration and the ethical guidelines for human genome and gene analysis research. Approval was obtained from the Ethics Committee of Tokyo Medical and Dental University (TMDU; Approval No. G2021-002) prior to conducting the study.

### ACTOnco+® assay

ACTOnco+® is a gene panel test that analyzes DNA for 440 genes and RNA for 31 fusion genes, providing comprehensive genomic insights. Beyond SNVs, indels, CNVs, and gene fusions, ACTOnco+® uniquely reports heterozygous deletions for specific haploinsufficient genes, enhancing its diagnostic capabilities. Quality control criteria were set at a minimum mean depth of 500× with target base coverage at 100× ≥ 85%. In addition, the test also employs RNA-based NGS to detect fusion transcripts from 31 genes Quality control criteria were set at a minimum of 20 000 total mapped reads, with at least 6 positive controls meeting this threshold.

Genomic DNA (40 ng) was amplified using 4 primer pair pools. Libraries were prepared with Ion AmpliSeq Library Kit (Thermo Fisher Scientific), and barcoded adaptors were added with Ion Amplicon Library Kit (Life Technologies). Barcoded libraries were attached to sequencing beads via emulsion PCR, and enriched using IonChef (Life Technologies). Library quality and quantity were assessed using a fragment analyzer (AATI) and Qubit (Invitrogen). The RNA integrity with more than or equal to 100 bp was used for analysis according to the manufacturer’s recommendations.

Sequencing was performed on the Ion Proton sequencer using the Ion PI chip (Life Technologies) according to the manufacturer’s protocol. Criteria for variant calling analysis included a minimum of 25 variant reads, with allele frequencies of at least 2% for actionable variants and 5% for others. Variants with a prevalence rate of more than 1% in Genome Aggregation r2.0.2 and ToMMo (Tohoku Medical Megabank Organization) database were excluded as polymorphisms.

Copy number variation (CNV) was analyzed using ONCOCNV.^8^ Aberration detection in tumor exome (ADTEx) was applied for baseline correction and tumor purity estimation using the change ratio of all loss of heterozygosity (LOH) and ASCNA in pooled single nucleotide polymorphisms data. CN amplification was defined as CN ≥ 6, gains as CN = 4 or 5, while homozygous and heterozygous deletions were defined as CN = 0 and CN = 1, respectively. Copy number loss estimation was omitted for samples with tumor purity <30%.

TMB was calculated using sequenced regions of ACTOnco®+. TMB estimates somatic nonsynonymous mutations per megabase of all protein-coding genes (whole exome). TMB result was reported as “TMB-High” (≥7.5 Muts/Mb), “TMB-Low” (<7.5 Muts/Mb), or “Cannot Be Determined” if sample purity is <30%.

Classification of MSI status is determined by a machine learning prediction algorithm. It uses >400 genomic loci with the changes in repeat numbers from a pooled microsatellite stable (MSS) baseline as features. The result is either MSS or MSI-H.

Assessment of clinical actionability was performed based on OncoKB and ACT Genomics in-house knowledge database. Mutations were categorized into 4 tiers based on their level of evidence, ranging from FDA-recognized or standard care biomarkers for FDA-approved drugs (levels 1 and 2) to plausible therapeutic benefits based on clinical trials, clinical cases, or preclinical studies (levels 3 and 4).

ACTOnco+® undergoes rigorous quality control measures to ensure the accuracy and reliability of the assay. It obtains CAP ACCREDITED, SNQ, and FDA clearance.

### FoundationOne® CDx assay

FoundationOne® CDx is a comprehensive genomic profiling assay that utilizes NGS technology. It covers the analysis of 324 genes and genomic regions, including single nucleotide variants (SNVs), insertions/deletions (indels), CNVs, and gene rearrangements.

FoundationOne® CDx utilizes a specific sequencing platform Illumina Hiseq 4000 (Illumina) for the analysis of genomic alterations. The raw sequencing data obtained from FoundationOne® CDx is processed and analyzed using bioinformatics pipelines and algorithms. The identified genetic alterations are annotated and classified based on established databases and curated knowledge bases. The results are then interpreted to provide clinically relevant information regarding potential targeted therapies, immunotherapies, and clinical trial eligibility.

FoundationOne® CDx undergoes rigorous quality control measures to ensure the accuracy and reliability of the assay. It has received regulatory approval as an in vitro diagnostic device and has been validated through extensive clinical studies.

The comprehensive genomic profiling provided by FoundationOne® CDx enables the identification of actionable genetic alterations in tumors, facilitating personalized treatment strategies and improving patient outcomes. The assay has been widely adopted in clinical practice to guide therapeutic decision-making and is recognized as a valuable tool in precision medicine for cancer patients.^9^

### Patients and samples

This study included patients who underwent FoundationOne® CDx at our institution between June 2019 and January 2022. The inclusion criteria were patients aged 20 years or older with solid tumors.

For patients who underwent FoundationOne® CDx before this study was initiated (retrospective cohort: between June 2019 and July 2021), informed consent was obtained either verbally or through an opt-out process. Archived FFPE tissue samples stored in the TMDU Biobank were utilized for genomic profiling using ACTOnco+®.

For patients who underwent FoundationOne® CDx after this study was started (prospective cohort: between August 2021 and January 2022), written consent was obtained. FFPE tissue samples were concurrently collected and prepared during the FoundationOne® CDx process for subsequent genomic profiling using ACTOnco+®. The turnaround time (TAT) was measured from the date of sample submission to the issuance of the clinical report.

### Expert panel

After the receipt of the clinical report, a multidisciplinary expert panel consisting of clinicians, pathologists, bioinformaticians, oncologists, radiologists, genetic counselors, and clinical research coordinators from TMDU reviewed the findings of the genetic alterations and determined the results regarding the suggested candidate drugs. The panel utilized their expertise and collective knowledge to assess the implications of the genomic profiling results in the context of each patient’s clinical situation.

### Analysis

To assess the agreement of actionable genetic alterations, we examined common gene variants in both assays, excluding somatic variants that were likely single nucleotide polymorphisms as reported in the ToMMo database, which contains sequencing data from 14 000 Japanese healthy individuals. As the variants detected in the ACTOnco+® test were aligned to GRCh37, the genomic coordinates were converted from GRCh37 to GRCh38 using NCBI Remap API (https://web.archive.org/web/20230406181227/, https://www.ncbi.nlm.nih.gov/genome/tools/remap/docs/api), and the variants contained in the following 2 ToMMo data sets were removed: tommo-14KJPN-20211208-GRCh38-gf-autosome.vcf and tommo-14KJPN-20211208-GRCh38-gf-chrX_PAR3.vcf.

In the evaluation of SNVs and indels, a comparative analysis was conducted on 257 genes, which were common targets for testing. Instances where SNVs and indels were exclusively identified by FoundationOne® CDx and not by ACTOnco+® prompted additional scrutiny, involving reference to the ACTOnco+® BAM file. Subsequent validation was undertaken through Sanger sequencing on remaining samples with adequate tumor cell content.

Amplification analysis encompassed 58 genes, while homozygous loss analysis was applied to 70 genes shared by both tests. For the frequently identified CDKN2A gene in CNV analysis, fundamental validation was executed through in situ hybridization (ISH).

MSI concordance was determined by comparing results for Microsatellite Status from each test. TMB assessment involved evaluating concordance rates for categories TMB ≥ 10 and TMB < 10 as reported by each test.

To address potential variations stemming from sample quality, a subset of cases with tumor cellularity exceeding 30% underwent analysis. Clinical reports served as the basis for an expert panel convened at TMDU to assess the number of actionable gene variants and recommended candidate drugs identified by each test.

All statistical analyses were performed with EZR ver. 1.6 (Saitama Medical Center, Jichi Medical University, Saitama, Japan), which is a graphical user interface for R (The R Foundation for Statistical Computing, Vienna, Austria). More precisely, it is a modified version of R commander designed to add statistical functions frequently used in biostatistics.^10^ Pearson’s correlation coefficients were calculated for copy number values and TMB values obtained from both tests. The disparity in actionable mutations and recommended candidate drugs was statistically evaluated using a paired t-test.

Interpretation of the results and manuscript writing were conducted by academic researchers at TMDU, and ACT Genomics was not involved.

## Results

### Patient characteristics

**Figure 1.**
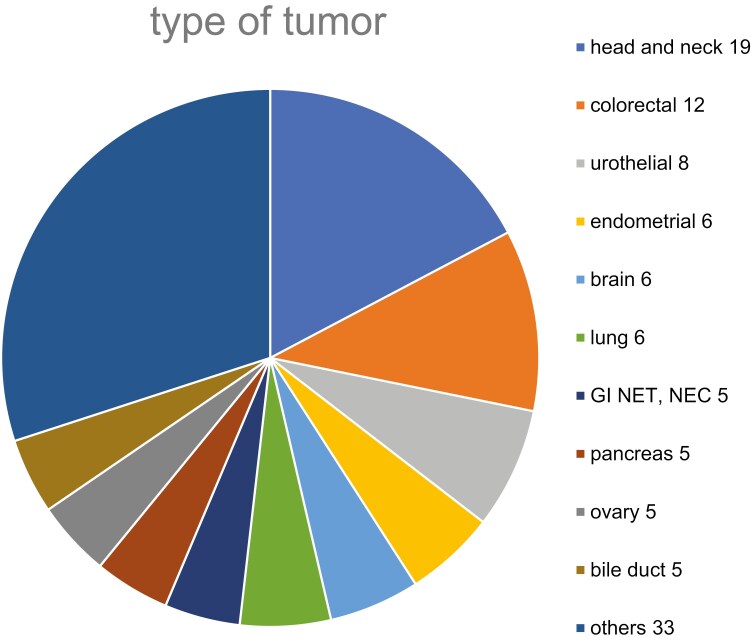
Types of tumors used in this study.

A total of 110 patients were included in the study, with 80 patients undergoing the FoundationOne® CDx between June 2019 and July 2021 (retrospective cohort), and an additional 30 patients undergoing the test between August 2021 and January 2022 (prospective cohort). Figure 1 illustrates the distribution of cases in the study. The median age at the time of testing was 65.5 years, with 61 male and 49 female patients. The most frequent tumor types were head and neck cancer (17.2%), colorectal cancer (10.9%), and urothelial carcinoma (7.2%). The median tumor cell percentage was 45%, and the median interval between sample collection and testing was 933.5 days (Table 1).

**Table 1.** Patient characteristics.

Age (median, years)	65.5
Sex (men/ female)	61/ 49
Types of tumors	
Head and neck	19 (17.2%)
Colorectal	12 (10.9%)
Urothelial	8 (7.2%)
Endmetrial	6 (5.4%)
Brain	6 (5.4%)
Lung	6 (5.4%)
GI NET, GI NEC	5 (4.5%)
Pancreas	5 (4.5%)
Ovary	5 (4.5%)
Bile duct	5 (4.5%)
Others**	33 (30.0%)
Tumor purity (median, range [%])	45% (range 30%-60%)
Years since specimen collection (median, days)	933.5 (range 545.5-1336.25)

** Others include Cervical cancer, Kidney cancer, Prostate cancer, Sarcoma, Malignant melanoma, Paget’s disease, Malignant peripheral nerve sheath tumor, Malignant mesothelioma, Stomach cancer, Liver cancer, Cancer of Unkown primary, Duodenal cancer, Small intestine cancer, Esophageal cancer, Breast cancer, Paraganglioma, Adrenal cancer.

### ACTOnco+® analysis

Among the 110 samples analyzed by ACTOnco+®, clinical-level testing was feasible for 109 samples. In one case, the clinical report was not available due to quality issues. The results of genetic testing, including the BAM file, were available for all 110 samples. The median TAT for ACTOnco+® was 17 days.

**Figure 2.**
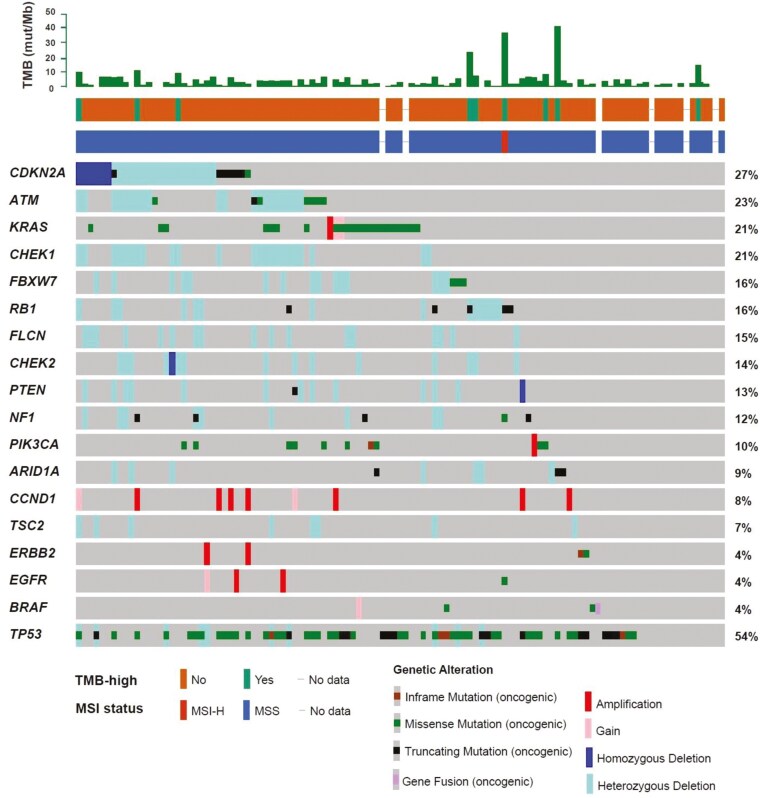
Landscape of oncogenic and actionable alterations detected with the ACTOnco+® assay. The Oncoprint displays the prevalence of oncogenic alterations in 110 patients.

The oncogenic variants detected in the ACTOnco+® clinical report are shown in Figure 2.^11,12^ The most frequently altered genes, in descending order, were *TP53* (54%), *CDKN2A* (27%), *ATM* (23%), *KRAS* (21%), and *CHEK1* (21%). For SNVs, the frequencies were as follows: *TP53* (52%), *KRAS* (20%), *PIK3CA* (9%), *APC* (7.2%), *CDKN2A* (7.2%), and *ERBB3* (7.2%). Amplifications were observed in the following genes, in descending order: *CCND1* (8%), *FGF3* (8%), *GNAS* (5%), *AURKA* (5%), and *MYC* (5%). Homozygous deletions were observed in *CDKN2A* (5%), *CHEK2, DICER1*, *FAT1*, *FH*, *PARP1*, *PTEN*, *PTPRT*, *SMAECB1*, and *TGFBR2* (each at 1%). Heterozygous deletions were observed in the following genes: *CHEK1* (21%), *ATM* (18%), *CDKN2A* (16%), *SMAD4* (15%), and *FLCN* (15%). Fusion alterations were detected in 3 cases, KIAA1549-BRAF fusion and TMPRSS2-ERG fusion, CD74-ROS1 fusion. Among the exclusive genes reported by ACTOnco+®, the frequencies of detection were as follows: *RAD50* (11%), *ERCC1* (6%), *FAT1* (5%), *TOP1* (4%), and *SERPINB3* (4%).

### Comparison between foundationOne® CDx and ACTOnco+®

Table 2 presents the comparative analysis of the results. For SNVs, the positive concordance rate was 87.9%, and the positive predictive value was 100.0%. We conducted a validation using the BAM file from ACTOnco+® to investigate 23 SNVs that were reported by FoundationOne® CDx, but not reported by ACTOnco+®. Among these 23 variants, 21 were identified within the BAM files of ACTOnco+®. After visual inspection, the positive agreement of SNV was 98.9%, positive predictive value was 100%. A total of 9 variants were examined by the sangar method, and 8 variants showed concordant results with FoundationOne CDx® (Supplementary Table 1).

**Table 2.** Comparison of variant detection.

SNVs		ACTOnco+®			
		Positive	Negative	Positive agreement	Positive predictive value
FoundationOne® CDx	positive	167	23	87.9%	100.0%
	negative	0	N/A		
**Indels**		ACTOnco+®			
		Positive	negative	positive agreement	positive predictive value
FoundationOne® CDx	positive	49	22	69.0%	96.1%
	negative	2	N/A		
**Amplification**		ACTOnco+®			
		positive	negative	positive agreement	positive predictive value
FoundationOne® CDx	positive	80	24	76.9%	77.7%
	negative	23	N/A		
**Homozygous loss**		ACTOnco+®			
		positive	negative	positive agreement	positive predictive value
FoundationOne® CDx	positive	18	9	66.7%	66.7%
	negative	9	N/A		
**MSI**		ACTOnco+®			
		MSI high	MSS	Cannot determined	
FoundationOne® CDx	MSI high	1	0	0	
	MSS	0	95	0	
	cannot determined	0	9	4	
**TMB**		ACTOnco+®			
		TMB ≧ 10	TMB < 10	cannot determined	
FoundationOne® CDx	TMB ≧ 10	4	4	0	
	TMB < 10	1	86	0	
	cannot determined	1	9	4	

In the case of indels, the positive concordance rate was 69.0%, and the positive predictive value was 96.1%. We performed a validation for 22 indel variants that were not reported by ACTOnco+®. The 16 variants were identified within the BAM files of ACTOnco+®. With visual inspection, the positive agreement of indel was 91.5%, positive predictive value was 97.2%.

For amplifications, the positive concordance rate was 76.9%, and the positive predictive value was 77.7%. For homozygous loss, the positive concordance rate was 66.7% and the positive predictive value was 66.7%. Further analysis with ISH on CDKN2A was performed on 16 variants with sufficient sample quantity and tumor cell content. The results of CN alteration by ACTOnco+® were in good concordance with the results of ISH (Supplementary Table 2).

Fusion genes were observed in 3 cases. TMPRSS2-ERG fusion and CD74-ROS1 fusion were detected in both FoundationOne® CDx and ACTOnco+®. KIAA1549-BRAF fusion was exclusively identified by ACTOnco+®.

**Figure 3.**
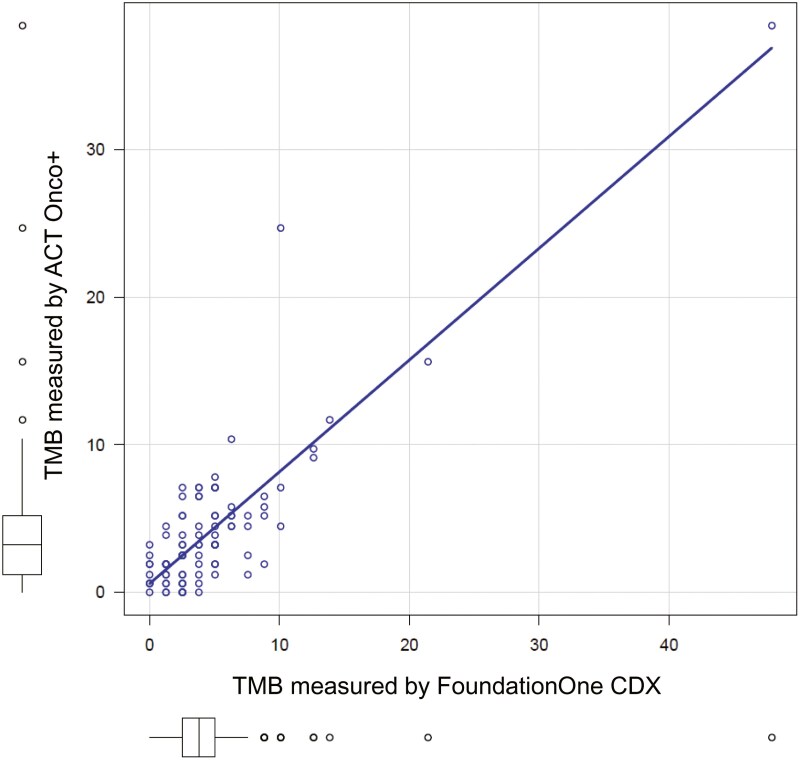
TMB (tumor mutation burden) comparison between FoundationOne CDx and ACT Onco + assays. R2 = 0.849, 95% Confidential Interval: 0.781-0.897, *P*-value < .0001

In terms of MSI and TMB, the results of both tests demonstrated preferable concordances. The TMB correlation coefficient of R = 0.849 (Figure 3).

In the subset with tumor cellularity exceeding 30%, a higher concordance rate was observed in SNVs (Supplementary Table 3). In the analysis of indels, a lower positive agreement rate of 61.0% was observed. For amplifications, positive agreement was 76.1%. For homozygous loss, an improved positive agreement rate of 78.2% was observed.

The number of actionable gene variants detected by ACTOnco+® were 6.80 per report, compared to 4.87 for FoundationOne® CDx per report (*P* < .01). The number of recommended candidate drugs per test report was 2.34 per report for ACTOnco+®, compared to 1.61 for FoundationOne® CDx (*P* < .01). In the analysis excluding heterozygous deletions, the number of actionable genes detected by ACTOnco+® was 3.82 per report (4.87 for FoundationOne® CDx, *P* < .01). The number of recommended candidate drugs per report was 1.49 for ACTOnco+® (1.61 for FoundationOne® CDx *P* = .11).

### A case study on the clinical utility of RNA sequencing with BRAF fusion

The patient’s medical history reveals that at the age of 9, a brain tumor was detected, leading to a surgical procedure involving cerebellar resection. Histopathological analysis confirmed the presence of pilocytic astrocytoma, and postoperative treatment consisted of carboplatin, etoposide, cyclophosphamide administration, and localized radiotherapy. The patient experienced tumor recurrence at the age of 10, which prompted subsequent interventions including local resection and temozolomide administration. Despite these efforts, the disease demonstrated a progressive trend. The identification of KIAA1549-BRAF fusion in the tumor specimen was achieved using ACTOnco+®, a comprehensive genomic profiling assay. No KIAA1549-BRAF was detected by FoundationOne CDx®. To validate the fusion gene detection, TSO500, another RNA sequencing test was used, confirming the presence of KIAA1549-BRAF fusion. Subsequently, the patient was enrolled in a clinical trial (The prospective trial of patient-proposed healthcare services with multiple targeted agents based on the result of gene profiling by multigene panel test [BELIEVE trial, jRCTs031190104]) investigating the efficacy of trametinib, a MEK inhibitor. The therapeutic agents were provided at no cost by Novartis Corporation. The administration of the therapeutic agent resulted in the maintenance of stable disease for more than 15 months (response is ongoing), indicating a favorable response to the treatment.

### A case study on the clinical utility of LOH in ATM with nonsense mutation and heterozygous deletion

The patient’s medical history reveals the initial diagnosis of medulloblastoma at the age of 15, leading to tumor resection surgery followed by localized radiotherapy and chemotherapy. Despite these interventions, the patient experienced tumor recurrence at the age of 25, leading to subsequent interventions including local resection and methotrexate administration. The disease demonstrated a progressive course, indicating the need for further investigation into potential underlying genetic factors. Genetic analysis identified the presence of ATM nonsense mutation and heterozygous deletion in the tumor specimen. These findings indicate a possible LOH event, leading to the inactivation of both alleles of ATM. This could be linked to heightened sensitivity to radiation, possibly explaining the unusually extended disease-free period following initial localized therapy.

## Discussion

In this study, we evaluated the results of ACTOnco+® in 110 patients who underwent FoundationOne® CDx at our institution. This study provides a direct comparison of DNA-only testing with DNA- and RNA-based testing.

For SNVs, a preferable concordance rate of 87.9% was observed. For indels, the positive concordance rate was 69.0% due to test characteristics. For amplifications, an overall concordance rate of 76.9% was observed, with a high correlation in individual copy number calls (Supplementary Figure 1). In cases with discrepancies, the copy numbers were below the cutoff in most instances. For homozygous loss, a concordance rate of 66.7% was observed. In some cases with discrepancies, heterozygous deletions were detected, but they were not within the reporting scope of ACTOnco+®. Non-detection was observed in cases with low tumor cellularity.

Among the 110 samples, 61 samples exhibited the detection of 329 heterozygous deletions. ISH was performed specifically for CDKN2A, showing a certain level of concordance with the ACTOnco+® findings. Although heterozygous deletion is believed to confer similar characteristics to truncation events in tumors, its clinical significance is still under investigation in terms of prognosis prediction and response rates. Haploinsufficient genes are a subset of genes that are sensitive to decreased gene dosage. Heterozygous deletion in haploinsufficient genes often leads to the LOH, which creates specific vulnerabilities in cancer cells. These vulnerabilities, also known as GEMINI vulnerabilities, represent potential therapeutic targets.^13,14^

Gene fusion events were detected in 3 cases. In one case, KIAA1549-BRAF fusion was identified by ACTOnco+®, leading to the inclusion of the patient in a clinical trial for MEK inhibitor. Numerous reports suggest a relatively high response rate to targeted therapies for fusion variants, such as BRAF and NTRK.^15-19^ DNA-based NGS assays face limitations in detecting gene fusions because they lack comprehensive breakpoint sequence data that covers clinically relevant, actionable gene fusions. RNA-based assays can detect functional fusion transcripts and provide data on expression levels of the fusion transcript and its isoforms.^20^

In terms of clinical utility, the ACTOnco+® panel detected an average of 6.8 actionable genes and identified an average of 2.34 potential therapeutic options (median). Overall, there was generally good concordance with the detection of the tested gene alterations. The reporting of heterozygous deletions for haploinsufficient genes contributed to the observed differences.

In this comparison, ACTOnco+® demonstrates efficacy by facilitating the detection of heterozygous deletions and gene fusions in a substantial proportion of tumors, potentially expanding the therapeutic strategy. However, this study is limited to a single institution and focused on clinical samples, which may introduce biases in terms of mutation prevalence, tumor types, and the limited number and quality of samples analyzed. Further studies on a larger scale involving a broader range of tumors are needed to deepen our understanding of the characteristics of this novel gene panel testing and its clinical implications.

## Conclusion

ACTOnco+® assay showed its unique capacity for RNA-based fusion gene analysis and reporting of heterozygous deletions in haploinsufficient genes. These distinctive features present unique features when compared to conventional methods.

## Supplementary Material

oyaf056_suppl_Supplementary_Tables_1-3

oyaf056_suppl_Supplementary_Figures_1

## Data Availability

The data underlying this article will be shared on reasonable request to the corresponding author.
