# Supplementary material for: Characterization of a novel comprehensive genomic profiling test with better detection of heterozygous deletions and RNA-based gene fusion analysis
Source: Oncologist. 2025 Aug 11;30(8):oyaf056. doi: 10.1093/oncolo/oyaf056 (PMC12342955; doi:10.1093/oncolo/oyaf056)
Supplement: oyaf056_suppl_Supplementary_Tables_1-3 [file oyaf056_suppl_supplementary_tables_1-3.docx]

**Supplementary Tables**

**Supplemental Table 1. Sanger sequencing validation of SNVs that was detected by FoundationOne® CDx, but not detected with ACTOnco+®.**

| study ID | position | gene | variant type | ACTOnco+® Bioinformatic  validation | Sanger  validation |
| --- | --- | --- | --- | --- | --- |
| 1 | chrX:53223519,G>T | KDM5C | nonsense | Detected | Mutation detected |
| 2 | chr13:48941720,C>T | RB1 | nonsense | Detected | Mutation detected |
| 3 | chr4:153247366,C>G | FBXW7 | missense | Detected | Mutation detected |
| 4 | chr9:21971120,G>A | CDKN2A | nonsense | Detected | Mutation detected |
| 5 | chr21:44524456,G>A | U2AF1 | missense | Detected | Mutation detected |
| 6 | chr15:88423598, T>G | NTRK3 | missense | Not detected | Mutation detected |
| 7 | chr4:106196248,delC | TET2 | Frameshift | Detected | Not detected |

**Supplemental Table 2. ISH validation**

| Study ID | ACT copy number | F1 copy number | FISH | tumor purity* | FISH agreement |
| --- | --- | --- | --- | --- | --- |
| 8 | 1 | 0 | 1 | 0.62 | ACT |
| 9 | 1 | 0 | 0 | 0.81 | F1CDx |
| 10 | 0 | 2 | 0 | 0.45 | ACT |
| 11 | 1 | 2 | 1 | 0.6 | ACT |
| 12 | 0 | 0 | 0 | 0.81 | Both |
| 13 | 2 | 0 | 0 | 0.3 | F1CDx |
| 14 | 0 | 0 | 1 | 0.65 | ACT |
| 15 | 2 | 0 | 0 | 0.3 | F1CDx |
| 16 | 1 | 0 | 0 | 0.36 | F1CDx |
| 17 | 1 | 2 | 0 | 0.39 | Neither |
| 18 | 1 | 0 | 1 | 0.47 | ACT |
| 19 | 2 | 0 | 0 | 0.3 | F1CDx |
| 20 | 1 | 2 | 1 | 0.51 | ACT |
| 21 | 2 | 0 | 0 | 0.3 | F1CDx |
| 22 | 1 | 2 | 1 | 0.78 | ACT |

* Tumor purity is measured in samples analyzed with ACTOnco+®. FoudationOne® CDx used samples from the same block, but accurate tumor fraction after sample processing is not available.

**Supplemental Table 3. Comparison of Variant Detection tumor purity >30%**

| **SNVs** |  | ACTOnco+® | |  |  |
| --- | --- | --- | --- | --- | --- |
|  |  | positive | negative | positive agreement | positive predictive value |
| FoundationOne® CDx | positive | 123 | 0 | 100% | 100.0% |
|  | negative | 0 | N/A |  |  |
|  |  |  |  |  |  |
| **Indels** |  | ACTOnco+® | |  |  |
|  |  | Positive | negative | positive agreement | positive predictive value |
| FoundationOne® CDx | positive | 36 | 23 | 61.0% | 94.7% |
|  | negative | 2 | N/A |  |  |
|  |  |  |  |  |  |
| **Amplification** |  | ACTOnco+® | |  |  |
|  |  | positive | negative | positive agreement | positive predictive value |
| FoundationOne® CDx | positive | 67 | 21 | 76.1% | 77.0% |
|  | negative | 20 | N/A |  |  |
|  |  |  |  |  |  |
| **Homozygous loss** |  | ACTOnco+® | |  |  |
|  |  | positive | negative | positive agreement | positive predictive value |
| FoundationOne® CDx | positive | 18 | 5 | 78.2% | 66.7% |
|  | negative | 9 | N/A |  |  |
|  |  |  |  |  |  |
| **MSI** |  | ACTOnco+® | | |  |
|  |  | MSI high | MSS | Cannot determined |  |
| FoundationOne® CDx | MSI high | 1 | 0 | 0 |  |
|  | MSS | 0 | 79 | 0 |  |
|  | cannot determined | 0 | 3 | 0 |  |
|  |  |  |  |  |  |
| **TMB** |  | ACTOnco+® | | |  |
|  |  | TMB≧ 10 | TMB <10 | cannot determined |  |
| FoundationOne® CDx | TMB≧ 10 | 3 | 3 | 0 |  |
|  | TMB < 10 | 1 | 72 | 0 |  |
|  | cannot determined | 0 | 4 | 0 |  |
