# Supplementary material for: Characterization of a novel comprehensive genomic profiling test with better detection of heterozygous deletions and RNA-based gene fusion analysis
Source: Oncologist. 2025 Aug 11;30(8):oyaf056. doi: 10.1093/oncolo/oyaf056 (PMC12342955; doi:10.1093/oncolo/oyaf056)
Supplement: oyaf056_suppl_Supplementary_Figures_1 [file oyaf056_suppl_supplementary_figures_1.pptx]

## Slide 1
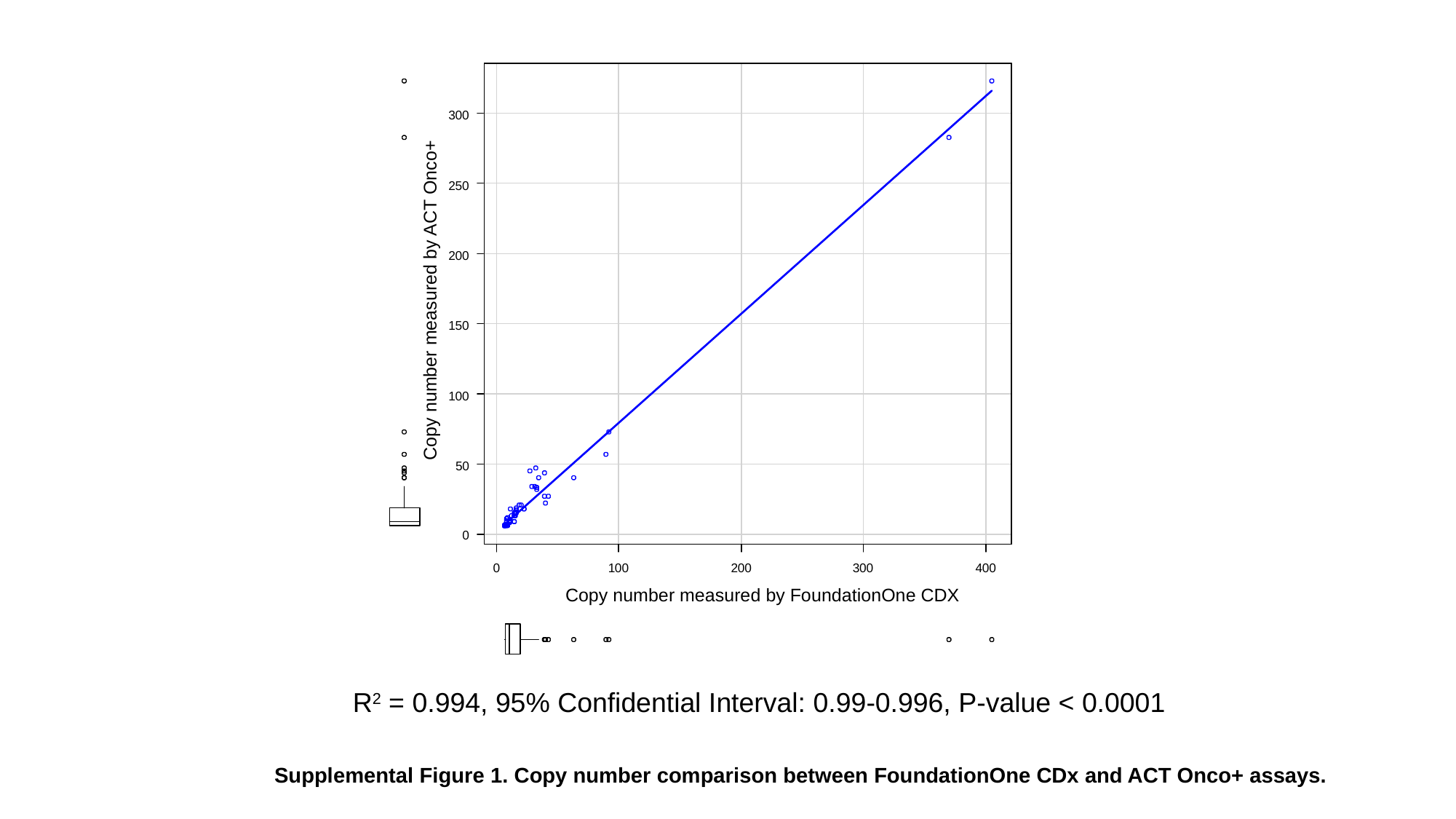

Copy number measured by ACT Onco+
Copy number measured by FoundationOne CDX
R2 = 0.994, 95% Confidential Interval: 0.99-0.996, P-value < 0.0001
Supplemental Figure 1. Copy number comparison between FoundationOne CDx and ACT Onco+ assays.
